# Prediction of various insulin resistance indices for the risk of hypertension among military young adults: the CHIEF cohort study, 2014–2020

**DOI:** 10.1186/s12933-024-02229-8

**Published:** 2024-04-25

**Authors:** Kun-Zhe Tsai, Chen-Chih Chu, Wei-Chun Huang, Xuemei Sui, Carl J. Lavie, Gen-Min Lin

**Affiliations:** 1 Department of Medicine, Hualien Armed Forces General Hospital, No. 100, Jinfeng St, Hualien City, 970 Taiwan; 2 Department of Stomatology of Periodontology, Mackay Memorial Hospital, Taipei, Taiwan; 3 Department of Periodontology, School of Dentistry, National Defense Medical Center and Tri-Service General Hospital, Taipei, Taiwan; 4 Department of Medicine, Tri-Service General Hospital and National Defense Medical Center, Taipei, Taiwan; 5 College of Medicine, National Yang Ming Chiao Tung University, Taipei, Taiwan; 6 Department of Critical Care Medicine, Kaohsiung Veterans General Hospital, Kaohsiung, Taiwan; 7 Department of Exercise Science, Arnold School of Public Health, University of South Carolina, Columbia, SC USA; 8 Ochsner Clinical School, John Ochsner Heart and Vascular Institute, The University of Queensland School of Medicine, New Orleans, LA USA

**Keywords:** Cohort study, Hypertension, Insulin resistance indices, Young adults

## Abstract

**Background:**

Non-insulin-based insulin resistance (NI-IR) indices have been reported to have an association with prevalent hypertension, however, no cohort studies to date have compared their prediction of hypertension among young adults.

**Methods:**

A total of 2,448 military men and women, aged 18–39 years, without baseline hypertension in Taiwan were followed for incident hypertension events from 2014 until the end of 2020. All subjects underwent annual health examinations including measurements of blood pressure (BP) in mmHg. Systolic BP (SBP) 130–139/diastolic BP (DBP) < 80, SBP < 130/DBP 80–89, and SBP 130–139/DBP 80–89 were respectively defined as stage I isolated systolic hypertension (ISH), isolated diastolic hypertension (IDH) and combined hypertension (CH). The cut-off levels of stage II hypertension for SBP and DBP were 140–159 and 90–99, respectively. Four NI-IR indices included the ratio of serum triglycerides (TG) to high-density lipoprotein cholesterol (HDL-C), TyG index defined as ln[TG* fasting glucose (FG)/2], Metabolic Score for IR (METS-IR) defined as ln[(2* FG) + TG)* body mass index (BMI)/(ln(HDL-C))], and ZJU index defined as BMI + FG + TG + 3* alanine transaminase/aspartate transaminase (+ 2 if female). Multivariable Cox regression analysis was performed with adjustments for baseline age, sex, body mass index, BP, substance use, family history for early onset cardiovascular diseases or hypertension, low-density lipoprotein cholesterol, kidney function, serum uric acid and physical activity to determine the associations.

**Results:**

During a median follow-up of 6.0 years, there were 920 hypertension events (37.6%). Greater TyG, TG/HDL-C and METS-IR indices were associated with a higher risk of stage I IDH (hazard ratios (HRs) and 95% confidence intervals: 1.376 (1.123–1.687), 1.082 (1.039–1.127) and 3.455 (1.921–6.214), respectively), whereas only greater ZJU index was associated with a higher risk of stage II IDH [HRs: 1.011 (1.001–1.021)]. In addition, greater ZJU index was associated with a higher risk of stage II ISH [HR: 1.013 (1.003–1.023)], and greater TyG index was associated with a higher risk of stage II CH [HR: 2.821 (1.244–6.395)].

**Conclusion:**

Insulin resistance assessed by various NI-IR indices was associated with a higher risk of hypertension in young adults, while the assessment ability for specific hypertension category may differ by NI-IR indices.

**Supplementary Information:**

The online version contains supplementary material available at 10.1186/s12933-024-02229-8.

## Introduction

Hypertension, widely acknowledged as a risk factor for chronic kidney disease, cardiovascular diseases (CVD), cognitive decline and dementia [1], remains a public health problem, despite the efforts of the scientific community [2]. Its global prevalence continues to trend upwards, expected to leap from roughly 972 million (26.4%) in 2000 to approximately 1.56 billion (29.2%) in 2025, marking a 60% increase over 25 years, which is partly due to worsening obesity and aging of the population [3]. According to a prospective study, hypertension contributes to a higher burden of mortality compared to any other identified risk factor [4]. In addition, hypertension-mediated organ damage is largely not reversible [5], emphasizing the critical importance of preventive measures.

Insulin resistance (IR) characterizes reduced sensitivity and responsiveness to insulin’s action, often manifesting several years prior to the onset of diabetes [6]. Mounting evidence suggests that IR and its related conditions significantly contribute to CVD development in both diabetic and non-diabetic populations [7]. IR serves as both a causative element and a marker of poor prognosis for individuals with CVD, regardless of their diabetic status.

Numerous studies have reported the link between IR and hypertension [8], indicating that IR may serve as a supplementary tool for assessing the potential risk of hypertension. However, only a few studies have included participants free of baseline hypertension before middle age. In addition, the hyperinsulinemic-euglycemic clamp, the gold standard for assessing IR, is invasive and time-consuming [9]; as a result, it is not commonly used for health screening or in epidemiological studies. Several non-insulin-based IR (NI-IR) indices have been proposed in the past decade [9–22]. Therefore, this study aimed to investigate the association between various NI-IR indices and new-onset hypertension in young adults.

## Methods

### Study population

The cardiorespiratory fitness and health in Eastern armed forces (CHIEF) study was conducted in Taiwan including 4,080 military men and women who were aged between 18 and 50 years at baseline in 2014. The participants were free of diabetes mellitus and did not take any medications, e.g., antihypertensive and lipid lowering medications at baseline [23]. This study was aimed to examine the correlates and associations of physical fitness and potential risk factors with cardiovascular and metabolic comorbidities in physically active young adults. At baseline, each participant underwent health evaluation, which encompassed various metrics, e.g., anthropometrics, hemodynamics, and blood biomarkers. The participants disclosed their third-degree relatives’ family history for early onset CVD or hypertension defined as the incidence occurring < 45 years in men and < 55 years in women [24]. In addition, the participants addressed substance use status, delineating alcohol intake and tobacco smoking as active and former/ never consumers, and their moderate-intensity physical activity (PA) levels through leisure-time running sessions, categorized as < 150 min per week, 150–299 min per week, and ≥ 300 min per week over the past six months. The information was obtained through a self-reported response to the questionnaire at the Hualien Armed Forces General Hospital.

The cohort study followed the ethical guidelines delineated in the Declaration of Helsinki. Additionally, the study design underwent evaluation and received approval from the Institutional Review Board (IRB) of the Mennonite Christian Hospital in Hualien City, Taiwan (No. 16-05-008). Prior to participation, written informed consent was acquired from all individuals involved in the study.

### Baseline health examinations (2014)

Each participant’s waist circumference (WC) and body height and weight were taken in standing position. The body mass index (BMI) was calculated as the ratio of the body weight in kilograms to square of the body height in square meters.

Fasting blood samples collected after a 12-hour overnight fast from each subject were used to determine serum concentrations of total cholesterol, low-density lipoprotein cholesterol (LDL-C), high-density lipoprotein cholesterol (HDL-C), triglycerides (TG), fasting plasma glucose (FG), serum uric acid (SUA), blood urea nitrogen (BUN), creatinine, alanine aminotransferase (ALT) and aspartate aminotransferase (AST). These biomarkers were analyzed using an automated analyzer (Olympus AU640, Kobe, Japan). Estimated glomerular filtration rate (eGFR) was calculated based on the 2021 Chronic Kidney Disease Epidemiology Collaboration (CKD-EPI) equation [25].

### Insulin resistance index calculation

In this study, four NI-IR indices for incident hypertension were compared. (1) The TG glucose (TyG) index was obtained from the formula: ln[TG (mg/dL) × FG (mg/dL)/2] [11]. (2) The TG to HDL-C ratio was derived by calculating TG (mg/dL) divided by HDL-C (mg/dL) [17]. (3) The metabolic score for insulin resistance (METS-IR) was calculated as ln[(2 × FG (mg/dL) + TG (mg/dL))× BMI/ ln[HDL-C (mg/dL)] [15]. (4) The Zhejiang University (ZJU) index was determined by the formula BMI + FG (mmol/L) + TG (mmol/L) + 3 × ALT (U/L)/AST (U/L) (+ 2, if female) [19].

### Definition of hypertension and phenotypes

Blood pressure (BP) was measured in a seated position using an oscillometric method though an automatic BP device (FT201 Parama-Tech Co., Ltd, Fukuoka, Japan) [26]. If the initial systolic/diastolic BP level exceeded 130/80 mmHg, a second measurement was taken after a 15-minute rest, and the final reported BP level was the average of both measurements [27].

According to the American College of Cardiology (ACC) and American Heart Association (AHA) guidelines [28], those with hypertension were defined as having a systolic BP (SBP) ≥ 130 mmHg and/or a diastolic BP (DBP) ≥ 80 mmHg. Isolated systolic hypertension (ISH) was defined as SBP ≥ 130 mmHg and DBP < 80 mmHg, isolated diastolic hypertension (IDH) as SBP < 130 mmHg and DBP ≥ 80 mmHg, and combined hypertension (CH) as SBP ≥ 130 mmHg and DBP ≥ 80 mmHg. Participants with a SBP 130–139 mmHg and/or DBP 80–89 mmHg were classified as having stage I hypertension. Those with SBP 130–139 mmHg and DBP < 80 mmHg were classified as stage I ISH, while SBP < 130 mmHg and DBP 80–89 mmHg were defined as stage I IDH. Those with SBP 130–139 mmHg and DBP 80–89 mmHg were classified as stage I CH. Participants with SBP 140–159 mmHg and/or DBP 90–99 mmHg were defined as having stage II hypertension. In addition, stage II ISH referred to SBP 140–159 mmHg and DBP < 90 mmHg, while stage II IDH referred to SBP < 140 mmHg and DBP 90–99 mmHg. Stage II CH was given to participants with SBP 140–159 mmHg and DBP 90–99 mmHg. Lifestyle modifications were recommended as the initial management for those developing stage I or stage II hypertension, and antihypertensive medications use was recommended as an adjuvant therapy to life style changes for those with stage II hypertension, consistent with standard of care during the early years of this study in both the United States and Europe.

### Statistical analysis

The baseline characteristics of the study cohort were presented as mean and standard deviation for continuous variables and as numbers and percentages for categorical variables. The follow-up for each participant started in 2014 and extended until the occurrence of hypertension events, loss to follow-up, or the end of the follow-up period, which ended on December 31, 2020.

The proportional hazards assumption was assessed by the Supremum test for the four NI-IR indicator variables in the cohort, separately (all p-values ≥ 0.20). Then, multivariable Cox hazards regression analysis was used to examine the hazard ratio (HR) and 95% confidence interval (CI) of each NI-IR index (every 1-unit increase) with the incidence of new-onset stage I, stage II and overall hypertension. Covariates used in the model were chosen based on previously published associations with hypertension [29–31]. If the correlation coefficient (r) between two covariates > 0.5 was found in Pearson correlation analysis, we selected the one which is not a MetS component or unrelated to a component of MetS while retained both SBP and DBP as they were the main essentials of hypertension (supplemental Table 1). SUA, a purine metabolite associated with both IR and hypertension in many previous studies [32–34] were also chosen as a potential covariate in this cohort study. For the primary analysis, the associations were initially adjusted for the demographic covariates (Model 1) and then for the blood biomarkers (Model 2). In Model 1, baseline age, sex, alcohol intake, tobacco smoking, PA levels, SBP, DBP, BMI and family history of early onset CVDs were initially adjusted for. In Model 2, LDL-C, SUA, BUN and eGFR were further adjusted for. The risk of incident ISH, IDH and CH associated with each NI-IR index was assessed in secondary analysis with adjustments for the covariates in Model 2.

The diagnostic performance of each NI-IR index to detect hypertension events was evaluated utilizing the area under the curves (AUC) of receiver operating characteristic (ROC). The sensitivity, specificity and optimal cut-off point of each IR index for hypertension events were calculated through ROC analysis. The performance using TyG index was treated as the reference and compared with the others by the Hanley and McNeil method [35]. Statistical significance was defined as a two-tailed p-value < 0.05. All statistical analyses were carried out using SPSS v26.0 for Windows, developed by IBM Corp. in Armonk, NY, USA.

## Results

### Study population and baseline characteristics

This study enrolled 4,080 participants at baseline. Of them, 1,024 subjects with hypertension, 58 subjects with age ≥ 40 years and 550 subjects lost to follow-up were excluded. Consequently, 2,448 participants with an average age of 28.15 ± 5.66 years were included for analysis. Of the final sample, 2,155 were male (88.0%), and the baseline SBP and DBP levels were 112.09 ± 10.31 mmHg and 66.50 ± 7.08 mmHg, respectively. Over a median follow-up period of 6.0 years, 920 participants (37.6%) developed new-onset hypertension, with 795 (32.5%) classified as stage I and 125 (5.1%) as stage II. No one had a SBP ≥ 160 mmHg and/or a DBP ≥ 100 mmHg during the follow-up period. Table 1 reveals further details about the baseline characteristics of the participants.

**Table 1:** Baseline characteristics of study cohort

	*N* = 2,448
Stage I hypertension	795 (32.5)
Isolated systolic hypertension, %	317 (12.9)
Isolated diastolic hypertension, %	346 (14.1)
Combined hypertension, %	132 (5.4)
Stage II hypertension	125 (5.1)
Isolated systolic hypertension, %	33 (1.3)
Isolated diastolic hypertension, %	71 (2.9)
Combined hypertension, %	21 (0.9)
Insulin resistance index	
TyG index	8.31 ± 0.55
TG/HDL-C	2.30 ± 2.03
METS-IR	2.01 ± 0.22
ZJU index	125.56 ± 15.54
Age, years	28.15 ± 5.66
Male sex, %	2155 (88.0)
Lifestyle behaviors	
Alcohol intake, %	961 (39.3)
Tobacco smoking, %	867 (35.4)
PA levels, %	
<150 min/wk	556 (22.7)
150–299 min/wk	931 (38.0)
≥300 min/wk	961 (39.9)
Family history of early onset CVD or hypertension, %	71 (2.9)
Systolic BP, mmHg	112.09 ± 10.31
Diastolic BP, mmHg	66.50 ± 7.08
Waist circumference, cm	81.48 ± 8.30
Body mass index, kg/m^2^	24.27 ± 3.09
18.5–24.9 kg/m^2^	1493 (61.0)
25.0–29.9 kg/m^2^	872 (35.6)
≥30.0 kg/m^2^	83 (3.4)
Blood test	
Total cholesterol, mg/dL	171.94 ± 33.05
LDL-C, mg/dL	103.57 ± 29.10
HDL-C, mg/dL	49.06 ± 10.31
TG, mg/dL	102.56 ± 69.90
FG, mg/dL	92.68 ± 12.76
Impaired fasting glucose, %	309 (12.6)
SUA, mg/dL	6.43 ± 1.37
BUN, mg/dL	12.61 ± 2.87
eGFR, mL/min/1.73m^2^	107.44 ± 12.82

Continuous variables are expressed as mean ± SD (standard deviation), and categorical variables as N (%)

Impaired fasting glucose is defined as fasting glucose of 100–125 mg/dL

Abbreviations: TyG, triglyceride glucose; TG, triglyceride; HDL-C, high-density lipoprotein cholesterol; METS-IR, metabolic score for insulin resistance; ZJU, Zhejiang University; PA, physical activity; BP, blood pressure; LDL-C, low-density lipoprotein cholesterol; FG, fasting plasma glucose; SUA, serum uric acid; BUN, blood urea nitrogen; CVD, cardiovascular disease; eGFR, estimated glomerular filtration rate

### NI-IR indices, stage I, stage II and overall hypertension

Table 2 shows the associations of various NI-IR indices with incident stage I, stage II and total hypertension. In the univariate analysis and in Model 1, all of the TyG, TG/HDL-C, METS-IR, and ZJU indices were linked to new-onset stage 2 hypertension across all categories. While additionally adjusting for the blood biomarkers in Model 2, only the TyG and ZJU indices were significantly associated with stage II hypertension [HRs and 95% CIs: 1.409 (1.024–1.939) and 1.010 (1.004–1.017), respectively]. Although the TG/HDL-C ratio and the METS-IR index were significantly associated with stage I hypertension [HRs: 1.037 (1.004–1.071) and 1.611 (1.085–2.392), respectively], stage 2 hypertension [HRs: 1.057 (1.002–1.15) and 2.998 (1.203–7.473), respectively] and total hypertension [HRs: 1.029 (1.002–1.56) and 1.594 (1.11–2.86), respectively] in Model 1, the associations of these two NI-IR indices with stage I, stage 2, and total hypertension were no longer statistically significant in Model 2.

**Table 2:** Association of insulin resistance index with new onset hypertension

Incidence of total hypertension								
	Crude model			Model 1			Model 2	
	HR (95% CI)	p-value		HR (95% CI)	p-value		HR (95% CI)	p-value
TyG index	1.804 (1.620–2.008)	< 0.001		1.135 (0.999–1.290)	0.052		1.083 (0.951–1.235)	0.22
TG/HDL-C	1.097 (1.076–1.118)	< 0.001		1.029 (1.002–1.056)	0.03		1.023 (0.995–1.052)	0.10
METS-IR	5.135 (3.893–6.774)	< 0.001		1.594 (1.111–2.286)	0.01		1.389 (0.958–2.014)	0.08
ZJU index	1.012 (1.010–1.015)	< 0.001		1.003 (0.999–1.007)	0.10		1.003 (0.999–1.008)	0.09
Incidence of stage I hypertension								
TyG index	1.716 (1.566–1.981)	< 0.001		1.102 (0.958–1.268)	0.17		1.049 (0.909–1.210)	0.51
TG/HDL-C	1.115 (1.088–1.141)	< 0.001		1.037 (1.004–1.071)	0.02		1.028 (0.995–1.063)	0.10
METS-IR	5.146 (3.783–6.999)	< 0.001		1.611 (1.085–2.392)	0.01		1.392 (0.927–2.090)	0.11
ZJU index	1.012 (1.009–1.014)	< 0.001		1.002 (0.997–1.007)	0.50		1.001 (0.996–1.006)	0.59
Incidence of stage II hypertension								
TyG index	2.907 (2.254–3.749)	< 0.001		1.527 (1.111–2.101)	0.009		1.409 (1.024–1.939)	0.03
TG/HDL-C	1.141 (1.101–1.183)	< 0.001		1.057 (1.002–1.115)	0.04		1.044 (0.987–1.105)	0.13
METS-IR	15 733 (8.195–30.205)	< 0.001		2.998 (1.203–7.473)	0.01		2.317 (0.904–5.938)	0.08
ZJU index	1.018 (1.014–1.023)	< 0.001		1.010 (1.004–1.017)	0.002		1.010 (1.004–1.017)	0.002

Data are presented as hazard ratios (HR) and 95% confidence intervals (CI) using multivariable Cox regression analysis

Model 1: adjust for age, sex, alcohol intake, tobacco smoking, family history for early-onset cardiovascular diseases or hypertension, physical activity levels, systolic blood pressure, diastolic blood pressure, and body mass index

Model 2: adjusted for variables in the Model 1 + low-density lipoprotein cholesterol, serum uric acid, blood urea nitrogen and estimated glomerular filtration rate

Abbreviations: AUC: area under curve; TyG, triglyceride glucose; TG, triglyceride; HDL-C, high-density lipoprotein cholesterol; METS-IR, metabolic score for insulin resistance; ZJU, Zhejiang University

### NI-IR indices and risk of specific hypertension phenotype

Table 3 shows the results of the multivariable-adjusted associations of various NI-IR indices with incident ISH, IDH and CH. The TyG, TG/HDL-C, METS-IR, and ZJU indices were associated with a higher risk of overall IDH [HRs: 1.355 (1.121–1.637), 1.060 (1.025–1.096), 3.006 (1.747–5.171), and 1.005 (1.000-1.010), respectively], rather than ISH and CH. The TyG index, TG/HDL-C, and METS-IR were associated with a higher risk of stage I IDH [HRs: 1.376 (1.123–1.687), 1.082 (1.039–1.127) and 3.455 (1.921–6.214), respectively], while no association found for stage I ISH and CH. Nevertheless, the association between the ZJU index and incident stage I IDH was borderline [HR: 1.005 (0.999–1.011), p-value = 0.09]. Moreover, the TyG index was linked to a higher risk of stage II CH [HR: 2.821 (1.244–6.395)], and the ZJU index demonstrated an association with incident stage II ISH and stage II IDH [HRs: 1.013 (1.003–1.023) and 1.011 (1.001–1.0021), respectively]. Both the TG/HDL-C and METS-IR were not associated with stage II ISH, stage II IDH and stage II CH.

**Table 3:** Association of insulin resistance index with new onset hypertension phenotype

Incidence of total hypertension								
	Isolated systolic hypertension			Isolated diastolic hypertension			Combined hypertension	
	HR (95% CI)	p-value		HR (95% CI)	p-value		HR (95% CI)	p-value
TyG index	0.835 (0.663–1.052)	0.12		1.355 (1.121–1.637)	0.002		1.056 (0.799–1.396)	0.70
TG/HDL-C	0.975 (0.915–1.040)	0.44		1.060 (1.025–1.096)	0.001		1.012 (0.942–1.087)	0.75
METS-IR	0.634 (0.326–1.233)	0.18		3.006 (1.747–5.171)	< 0.001		1.624 (0.716–3.683)	0.24
ZJU index	0.995 (0.985–1.005)	0.30		1.005 (1.000–1.010)	0.04		1.005 (0.997–1.013)	0.23
Incidence of stage I hypertension								
TyG index	0.826 (0.653–1.046)	0.11		1.376 (1.123–1.687)	0.002		0.828 (0.575–1.193)	0.31
TG/HDL-C	0.975 (0.913–1.041)	0.44		1.082 (1.039–1.127)	< 0.001		0.964 (0.873–1.065)	0.46
METS-IR	0.619 (0.313–1.221)	0.16		3.455 (1.921–6.214)	< 0.001		1.196 (0.425–3.366)	0.73
ZJU index	0.995 (0.984–1.005)	0.31		1.005 (0.999–1.011)	0.09		0.995 (0.980–1.009)	0.47
Incidence of stage II hypertension								
TyG index	1.643 (0.892–3.027)	0.11		1.265 (0.825–1.939)	0.28		2.821 (1.244–6.395)	0.01
TG/HDL-C	1.074 (0.931–1.240)	0.32		1.049 (0.974–1.130)	0.20		1.161 (0.963–1.398)	0.11
METS-IR	4.216 (0.689–25.80)	0.11		2.179 (0.622–7.628)	0.22		6.940 (0.617–78.10)	0.11
ZJU index	1.013 (1.003–1.023)	0.009		1.011 (1.001–1.021)	0.03		1.010 (0.982–1.038)	0.49

Data are presented as hazard ratios (HR) and 95% confidence intervals (CI) using multivariable Cox regression analysis for age, sex, alcohol intake, tobacco smoking, family history for early-onset cardiovascular diseases or hypertension, body mass index, physical activity levels, systolic blood pressure, diastolic blood pressure, low-density lipoprotein cholesterol, serum uric acid, blood urea nitrogen and estimated glomerular filtration rate adjustments

Abbreviations: TyG, triglyceride glucose; TG, triglyceride; HDL-C, high-density lipoprotein cholesterol; METS-IR, metabolic score for insulin resistance; ZJU, Zhejiang University

### Diagnostic performance of NI-IR indices

**Fig. 1:**
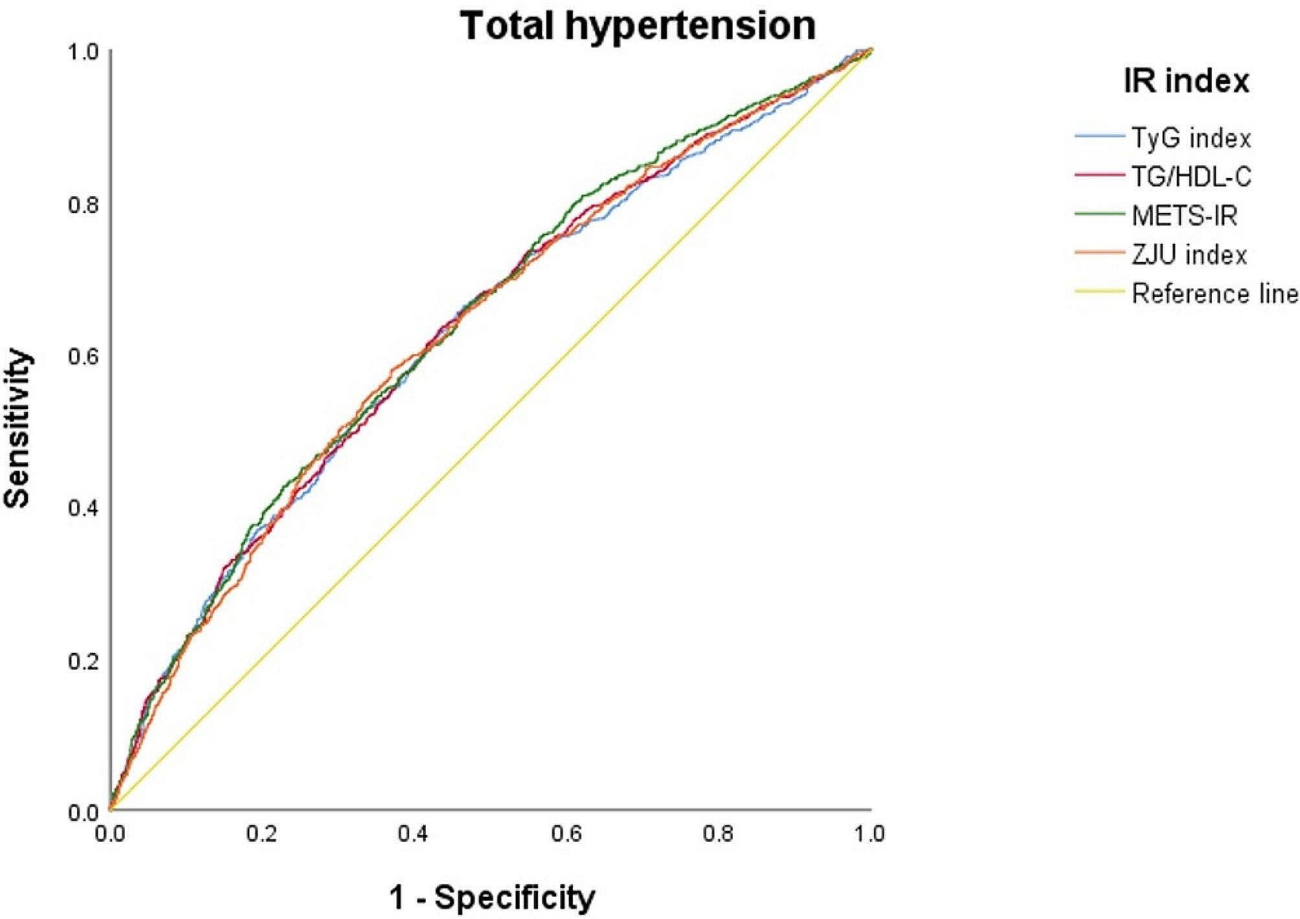
The area under the curves of receiver operating characteristic (AUC- ROC) for the four non-insulin based insulin resistance (NI-IR) indices to predict incident overall hypertension defined by blood pressure ≥ 130/80 mmHg

**Fig. 2:**
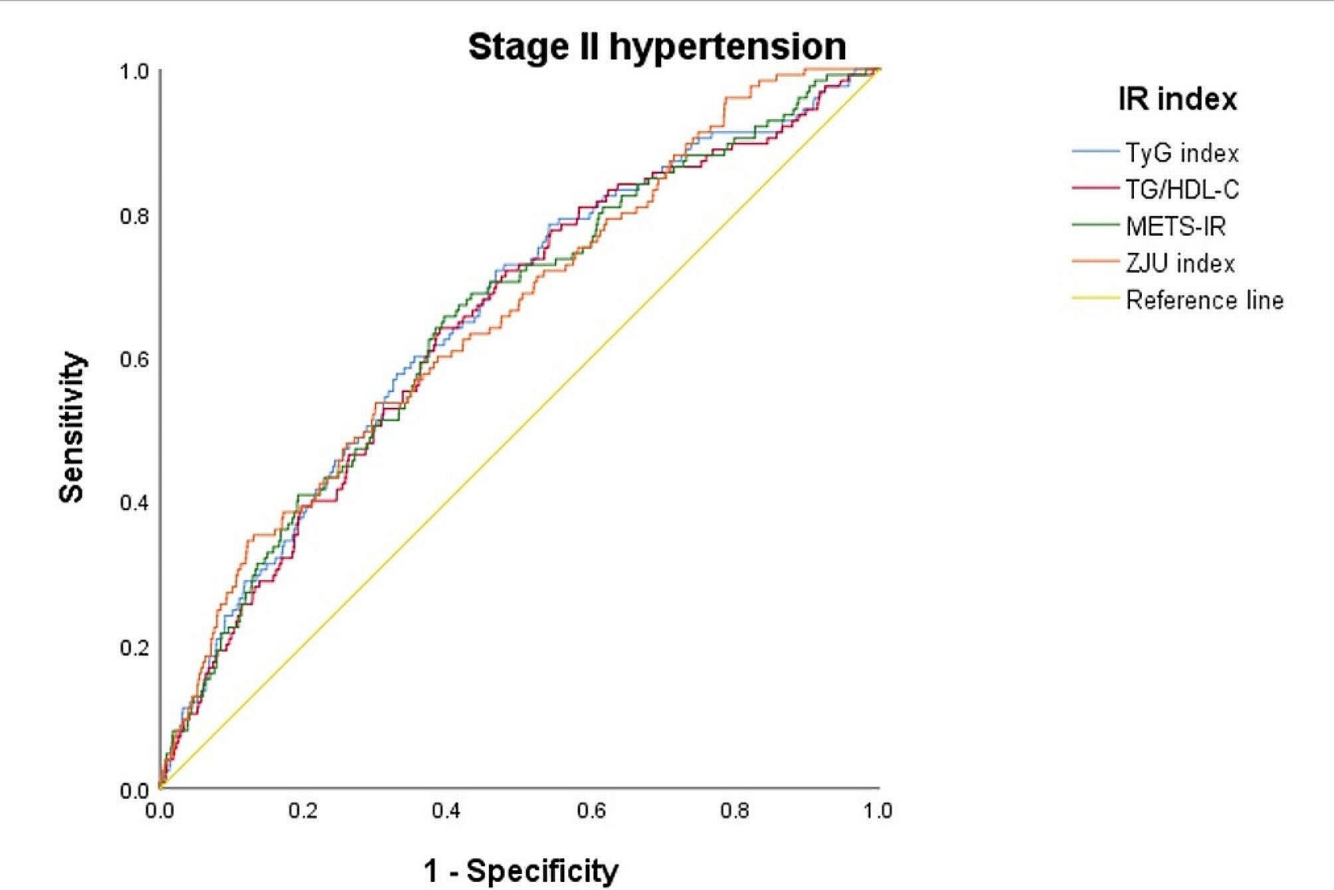
The AUC-ROC for the four non-insulin based insulin resistance (NI-IR) indices to predict incident stage II hypertension defined by blood pressure 140–159/90–99 mmHg

The diagnostic performance of various insulin resistance indices for the risk of incident stage II and overall hypertension are compared in Figs. 1 and 2, respectively. Figure 1 reveals the AUCs under the ROC curve ranged from 0.627 (0.604–0.650) for the TyG index to 0.641 (0.618–0.663) for the METS-IR. No significant differences were found among the four indices. Figure 2 reveals the AUCs under the ROC curve ranged from 0.687 (0.637–0.736) for the TG/HDL ratio to 0.695 (0.646–0.744) for the TyG index. No differences among the four indices were found as well. In Table 4, we identified the cut-off point for each index to yield the best outcome assessed by sensitivity and specificity to predict the development of overall new-onset hypertension from Fig. 1 and stage II hypertension from Fig. 2.

**Table 4:** Receiver operating characteristic (ROC) curve analysis for incidence of hypertension

Incidence of total hypertension						
	Cutoff point	Sensitivity	Specificity	AUC	95% CI	p-value
TyG index	8.199	0.654	0.545	0.627	0.604–0.650	reference
TG/HDL-C	1.693	0.634	0.566	0.631	0.608–0.654	0.85
METS-IR	1.963	0.664	0.535	0.641	0.618–0.663	0.44
ZJU index	125.223	0.584	0.627	0.631	0.608–0.653	0.85
Incidence of stage II hypertension						
TyG index	8.270	0.720	0.591	0.695	0.646–0.744	reference
TG/HDL-C	2.018	0.640	0.665	0.686	0.637–0.736	0.82
METS-IR	2.035	0.656	0.663	0.691	0.642–0.740	0.90
ZJU index	128.991	0.536	0.755	0.692	0.645–0.739	0.93

With age, sex, alcohol intake, tobacco smoking, family history for early-onset cardiovascular diseases or hypertension, physical activity levels, systolic blood pressure, diastolic blood pressure, body mass index, low-density lipoprotein cholesterol, serum uric acid, blood urea nitrogen and estimated glomerular filtration rate adjustments

Abbreviations: AUC: area under curve; TyG, triglyceride glucose; TG, triglyceride; HDL-C, high-density lipoprotein cholesterol; METS-IR, metabolic score for insulin resistance; ZJU, Zhejiang University

## Discussion

Our main findings suggest that among young adults, all four NI-IR indices were positively associated with incident hypertension, particularly in identifying IDH. The assessment ability for specific hypertension category may differ by NI-IR indices. For instance, the ZJU index revealed predictability in assessing the risk of stage II ISH, while the TyG index could be utilized for assessing the risk of stage II CH among young adults. In addition, no significant differences were observed for the diagnostic performance in overall and stage II hypertension among the four NI-IR indices.

The TyG index exhibits a good correlation with homeostatic model assessment (HOMA-IR) and is acknowledged as the preferred method for assessing IR in hypertensive Asian individuals [9]. In recent years, there has been extensive discussion on the correlation between TyG index and the risk of hypertension. Our findings align with previous cohort studies, affirming that elevated TyG index levels are associated with a higher risk of developing hypertension defined as BP ≥ 140/90 mmHg [12, 13, 16, 18, 36]. A recent meta-analysis encompassing 8 studies and 200,044 adults also revealed a higher risk of hypertension defined the same way in the highest TyG index compared to the lowest one [21]. However, some cross-sectional studies did not find any relationship between TyG index and hypertension defined as BP ≥ 140/90 mmHg or ≥ 130/85 mmHg in both obese and normal-weight individuals [10, 14].

To date, only cross-sectional studies were available to investigate the connection between TG/HDL-C and hypertension. A study, including 112,798 participants in China, revealed an association between TG/HDL-C and a higher prevalence of hypertension defined as BP ≥ 140/90 mmHg [17]. This relationship was not dose-dependent and marked by a diminishing slope of the curve as TG/HDL-C levels increased [17]. Findings from another study suggest that although the TG/HDL-C ratio was associated with prevalent hypertension defined as BP ≥ 140/90 mmHg, while its predictive efficacy fell short of WC or BMI [37]. Conversely, a study enrolling 142,005 adults showed no association between TG/HDL -C and hypertension defined as BP ≥ 130/85 mmHg [14].

To the best of our knowledge, no study has investigated the relationship between ZJU index and risk of hypertension. As the ZJU index was initially developed to correlate to the severity of IR and nonalcoholic steatohepatitis [19] which was associated with the occurrence of hypertension [38], it is reasonable that there was a link between ZJU index and new-onset hypertension in young adults. With regard to the METS-IR index, a recent meta-analysis comprising 8 cohort studies and 305,341 adults demonstrated that elevated METS-IR is linked to hypertension in the general population of adults [39]. In prior cohort studies, the association between METS-IR and hypertension was established, regardless of the BP levels defined for hypertension [20, 40]. In this study for young military adults, METS-IR was associated with the risk of stage I, stage II and overall hypertension, and specifically with new-onset IDH. In the BP trajectory, individuals with untreated hypertension experience a progressively increase in SBP throughout their life; while DBP would gradually increase until the age of 50 but decline sixth decade forward [41, 42]. IDH results from an increase in peripheral vascular resistance and is more prevalent in young and middle-aged adults [43, 44], prominently in the Asian population [45]. IDH has been well acknowledged as a risk factor of the development of CVD [46].

Although the mechanisms linking IR to the development of hypertension remain unclear, several pathways have been proposed. One mechanism suggests that hyperinsulinemia closely related to IR may activate the renin-angiotensin-aldosterone system, promote renal sodium retention [47] and ultimately lead to hypertension [22]. Another possible pathway is that IR may stimulate the sympathetic nervous system, resulting in the release of adrenaline and norepinephrine. This cascade effect contributes to increased cardiac output and peripheral vascular tone, which are facilitated by vascular smooth muscle cell hypertrophy and endothelial dysfunction [48].

### Strengths and limitations

There were some strengths in this study. First, this study was the first report to compare the associations between various NI-IR indices with the incidence of hypertension in young adults. In addition, all of the participants received at least one follow-up visit for the BP measurements during the study period. Nevertheless, this study had some limitations. First, this is an observational study that the cause-effect relationship between various NI-IR indices and hypertension could not be confirmed. Second, since this study was carried out exclusively for the young and physically fit military personnel in Taiwan who were characterized by predominantly male individuals (88%), lower WC and BMI levels compared to global averages and a lower WC than that in age- and sex-matched young adults from the general population in Taiwan [49], it is difficult to apply the results of this study to the general population, including the elderly and female individuals. Future studies are needed to include more women and a more diverse age range to enhance the generalizability of the conclusions. Third, as we lacked the information on fasting insulin, the risk of incident hypertension with HOMA-IR could not be available for a comparison with the proposed four NI-IR indices. Fourth, since the incidence of overall hypertension (*N* = 930, 37.6%) using the latest U.S. criteria (≥ 130/80 mmHg) in our young adults was much higher than the global average and the incidence of hypertension (*N* = 125, 5.1%) using the prior U.S. criteria (≥ 140/90 mmHg), the prevalence of hypertension using the latest U.S. criteria in an independent sample of 4,973 Taiwanese military personnel who had similar characteristics including the age ranges of 26–45 years, relevant to that in this study cohort at the end of follow-up was estimated to 33.2% which was close to the incidence of hypertension (37.6%) in this study cohort. Finally, although we verified the new-onset hypertension by an average of two independent measurements of resting BP at follow-up visits, the protocol regarding the BP measurement for each participant was not in line with the latest U.S. guideline, and there were no information on the existence of secondary hypertension, which might not be related to IR and thus resulted in a bias.

## Conclusion

Our findings suggest that IR as assessed by the TyG, TG/HDL-C, METS-IR, and ZJU indices was positively associated with the incidence of hypertension in young, relatively healthy and active adults. The assessment for the specific types of hypertension may need using different NI-IR indices. It is notable that the positive association between all four NI-IR indices and incident IDH was found, indicating a strong link between IR and peripheral arterial resistance even in young adults who would generally be considered at low risk of CVD.

### Electronic supplementary material

Below is the link to the electronic supplementary material.

Supplementary Material 1

## Data Availability

The datasets generated and/or analyzed during the current study are not publicly available due to materials obtained from the military in Taiwan, which were confidential, but are available from the corresponding author on reasonable request.

## Abbreviations

ACC: American college of cardiology
AHA: American heart association
ALT: Alanine aminotransferase
AST: Aspartate aminotransferase
AUC: Area under curve
BMI: Body mass index
BP: Blood pressure
CH: Combined hypertension
CHIEF: Cardiorespiratory fitness and health in Eastern armed forces
CI: Confidence intervals
DBP: Diastolic blood pressure
eGFR: Estimate glomerular filtration rate
FG: Fasting plasma glucose
HDL-C: High-density lipoprotein cholesterol
HOMA: Homeostatic model assessment
HR: Hazard ratio
IDH: Isolated diastolic hypertension
ISH: Isolated systolic hypertension
IR: Insulin resistance
IRB: Institutional review board
LDL-C: Low-density lipoprotein cholesterol
MDRD: Modification of diet in renal disease
MEST-IR: Metabolic score for insulin resistance
NHANES: National health and nutrition examination survey
NI-IR: Non-insulin-based insulin resistance
PA: Physical activity
PEACE: Patient-centered evaluation assessment of cardiac events
ROC: Receiver operating characteristic
SBP: Systolic blood pressure
SD: Standard deviation
TG: Triglyceride
TyG: Triglyceride glucose
WC: Waist circumference
ZJU: Zhejiang university
