# Supplementary material for: Prediction of various insulin resistance indices for the risk of hypertension among military young adults: the CHIEF cohort study, 2014–2020
Source: Cardiovasc Diabetol. 2024 Apr 25;23:141. doi: 10.1186/s12933-024-02229-8 (PMC11046748; doi:10.1186/s12933-024-02229-8)
Supplement: Supplementary file 1 — Supplementary Material 1 [file 12933_2024_2229_MOESM1_ESM.docx]

**Supplemental Table 1.** Pearson correlations between each potential exposure variables including four NI-IR indices

|  | Age | Sex | Alcohol intake | Tobacco smoking | PA levels | Family history | SBP | DBP | WC | BMI | TC | LDL-C | UA | BUN | eGFR | TyG index | TG/HDL-C | METS-IR | ZJU index |
| --- | --- | --- | --- | --- | --- | --- | --- | --- | --- | --- | --- | --- | --- | --- | --- | --- | --- | --- | --- |
| Age | 1.000 | -0.092* | 0.125* | 0.041* | 0.021 | 0.124* | 0.017 | 0.225* | 0.298* | 0.252* | 0.304* | 0.254* | 0.009 | 0.042* | -0.402* | 0.348* | 0.240* | 0.280* | 0.253* |
| Sex | -0.092* | 1.000* | -0.162* | -0.185* | -0.109* | 0.026 | -0.248* | -0.136* | -0.362* | -0.212* | -0.055* | -0.132* | -0.464* | -0.251* | 0.238* | -0.184* | -0.156* | -0.256* | -0.179* |
| Alcohol intake | 0.125* | -0.162* | 1.000 | 0.366* | -0.016 | 0.041* | 0.049* | 0.053* | 0.153* | 0.109* | 0.068* | 0.055* | 0.127* | 0.065* | -0.093* | 0.126* | 0.086* | 0.110* | 0.117* |
| Tobacco smoking | 0.041* | -0.185* | 0.366* | 1.000 | -0.025 | 0.013 | 0.018 | -0.011 | 0.079* | 0.026 | -0.005 | 0.031 | 0.072* | -0.008 | -0.051* | 0.135* | 0.131* | 0.167* | 0.055* |
| PA levels | 0.021 | -0.109* | -0.016 | -0.025 | 1.000 | -0.034 | 0.081* | 0.036 | 0.044* | 0.054* | -0.004 | -0.003 | 0.047* | 0.064* | -0.042* | -0.011 | -0.026 | 0.000 | 0.009 |
| Family history | 0.124* | 0.026 | 0.041* | 0.013 | -0.034 | 1.000 | -0.035 | 0.000 | 0.013 | 0.002 | 0.038 | 0.016 | -0.017 | -0.015 | 0.003 | 0.079* | 0.091* | 0.056* | 0.088* |
| SBP | 0.017 | -0.248* | 0.049* | 0.018 | 0.081* | -0.035 | 1.000 | 0.531* | 0.268* | 0.243* | 0.065* | 0.072* | 0.160* | 0.018 | -0.050* | -0.150* | 0.103* | 0.155* | 0.171* |
| DBP | 0.225* | -0.136* | 0.053* | -0.011 | 0.036 | 0.000 | 0.531* | 1.000 | 0.193* | 0.174* | 0.136* | 0.115* | 0.110* | 0.006 | -0.149* | 0.184* | 0.108* | 0.140* | 0.173* |
| WC | 0.298* | -0.362* | 0.153* | 0.079* | 0.044* | 0.013 | 0.268* | 0.193* | 1.000 | 0.841* | 0.278* | 0.316* | 0.394** | 0.157* | -0.197* | 0.403* | 0.340* | 0.515* | 0.429* |
| BMI | 0.252* | -0.212* | 0.109* | 0.026 | 0.054* | 0.002 | 0.243* | 0.174* | 0.841* | 1.000 | 0.264* | 0.298* | 0.336* | 0.132* | -0.168* | 0.386* | 0.324* | 0.517* | 0.455* |
| TC | 0.304* | -0.055* | 0.068* | -0.005 | -0.004 | 0.038 | 0.065* | 0.136* | 0.278* | 0.264* | 1.000 | 0.886* | 0.170* | 0.102* | -0.207* | -0.420* | 0.274* | 0.243* | 0.265* |
| LDL-C | 0.254* | -0.132* | 0.055* | 0.031 | -0.003 | 0.016 | 0.072* | 0.115* | 0.316* | 0.298* | 0.886* | 1.000 | 0.220* | 0.100* | -0.189* | 0.307* | 0.159* | 0.295* | 0.200* |
| SUA | 0.009* | -0.464* | 0.127* | 0.072* | 0.047* | -0.017 | 0.160* | 0.110* | 0.394* | 0.336* | 0.170* | 0.220* | 1.000 | 0.217* | -0.287* | 0.237* | 0.215* | 0.316* | 0.191* |
| BUN | 0.042* | -0.251* | 0.065* | -0.008 | 0.064* | -0.015 | 0.018 | 0.006 | 0.157* | 0.132* | 0.102** | 0.100* | 0.217* | 1.000 | -0.285* | 0.014 | 0.018 | 0.037 | 0.077* |
| eGFR | -0.402* | 0.238* | -0.093* | -0.051* | -0.042* | 0.003 | -0.050* | -0.149* | -0.197* | -0.168* | -0.207* | -0.189* | -0.287* | -0.285* | 1.000 | -0.201* | -0.141* | -0.189* | -0.120* |
| TyG index | 0.348* | -0.184* | 0.126* | 0.135* | -0.011 | 0.079* | 0.150* | 0.184* | 0.403* | 0.386* | 0.420* | 0.307* | 0.237* | 0.014 | -0.201* | 1.000 | 0.833* | 0.837* | 0.620* |
| TG/HDL-C | 0.240* | -0.156* | 0.086* | 0.131* | -0.026 | 0.091* | 0.103* | 0.108* | 0.340* | 0.324* | 0.274* | 0.159* | 0.215* | 0.018 | -0.141* | 0.833* | 1.000 | 0.840* | 0.482* |
| METS-IR | 0.280* | -0.256* | 0.110* | 0.167* | 0.000 | 0.056* | 0.155* | 0.140* | 0.515* | 0.517* | 0.243* | 0.295* | 0.316* | 0.037 | -0.189* | 0.837** | 0.840* | 1.000 | 0.495* |
| ZJU index | 0.253* | -0.179* | 0.117* | 0.055* | 0.009 | 0.088* | 0.171* | 0.173* | 0.429* | 0.455* | 0.256* | 0.200* | 0.191* | 0.077* | -0.120* | 0.620** | 0.482* | 0.495* | 1.000 |

Abbreviations: BMI, body mass index; TyG, triglyceride glucose; TC, total cholesterol; HDL-C, high-density lipoprotein cholesterol; METS-IR, metabolic score for insulin resistance; ZJU, Zhejiang University; PA, physical activity; LDL-C, low-density lipoprotein cholesterol; FG, fasting glucose; SUA, serum uric acid; BUN, blood urea nitrogen; eGFR, estimated glomerular filtration rate; WC, waist circumference; SBP, systolic blood pressure; DBP, diastolic blood pressure

* p <0.05
